# Local false discovery rate estimation using feature reliability in LC/MS metabolomics data

**DOI:** 10.1038/srep17221

**Published:** 2015-11-24

**Authors:** Elizabeth Y. Chong, Yijian Huang, Hao Wu, Nima Ghasemzadeh, Karan Uppal, Arshed A. Quyyumi, Dean P. Jones, Tianwei Yu

**Affiliations:** 1 Department of Biostatistics and Bioinformatics, Rollins School of Public Health, Emory University, Atlanta, GA, USA, 30322; 2 Department of Medicine, School of Medicine, Emory University, Atlanta, GA, USA, 30322

## Abstract

False discovery rate (FDR) control is an important tool of statistical inference in feature selection. In mass spectrometry-based metabolomics data, features can be measured at different levels of reliability and false features are often detected in untargeted metabolite profiling as chemical and/or bioinformatics noise. The traditional false discovery rate methods treat all features equally, which can cause substantial loss of statistical power to detect differentially expressed features. We propose a reliability index for mass spectrometry-based metabolomics data with repeated measurements, which is quantified using a composite measure. We then present a new method to estimate the local false discovery rate (lfdr) that incorporates feature reliability. In simulations, our proposed method achieved better balance between sensitivity and controlling false discovery, as compared to traditional lfdr estimation. We applied our method to a real metabolomics dataset and were able to detect more differentially expressed metabolites that were biologically meaningful.

High-throughput biological data, such as gene expression, proteomics and metabolomics data, presents large amounts of information with tens of thousands of features detected and quantified in complex biological matrices. In metabolomics, many features can represent the same metabolite, due to isotopes, adducts, in-source fragments or multiple-charged species. In addition to these, redundant features associated with the same metabolite, many features can be artifacts caused by chemical and/or bioinformatics noise. The aim is often to reduce the high-dimensional data by filtering out the false features and identifying a small group of true biomarkers. Here, features refer to individual entities measured in the specific type of biological data, e.g. genes, proteins, metabolites, while biomarkers are features whose levels change with respect to a clinical outcome or stage of a disease and are crucial to early diagnosis of disease and prognosis of treatment. Accurate selection of biomarkers is important for further validation studies, analysis of biological mechanisms, and building predictive models. The analysis of high-throughput data requires simultaneous hypothesis tests of each feature’s association with certain clinical outcomes. This creates the well-known multiple testing problem, and creates difficulties in statistical inference and data interpretation.

The concept and estimation procedures of False Discovery Rate (FDR) was developed to address this multiplicity issue^1^,^2^, which provides a sound statistical framework for inference and feature selection. The FDR is the expected proportion of falsely rejected null hypotheses, i.e. false discoveries, among all features called significant. The local false discovery rate (lfdr, in contrast to global FDR proposed by Benjamini and Hochberg, 1995) extends the concept of FDR to give a posterior probability at the single feature level^3^, i.e. the probability a specific feature being null given the test statistics of all features in the study.

Over the years, a number of estimation procedures were developed for FDR and lfdr^2^,^4^,^5^,^6^,^7^,^8^,^9^,^10^,^11^. Much effort has been invested in the estimation of the null distribution and proportion of differentially expressed features. Although different modeling approaches were used, all the methods share some common theme – the features are treated equally, certain statistics or p-values are computed for each feature, and the false discovery rates are computed based on the estimation of the distribution of null density from the observed test statistics or p-values.

In many high-throughput datasets, and especially with metabolomics, features are measured at different reliability levels. Here by “reliability” we refer to the confidence level we have on the point estimates of the expression values of a feature. In statistical terms, it can mean the size of the confidence interval relative to the measured values, which has a direct bearing on the statistical power to detect differential expression of the feature. In some other situations, it can also mean the probability that a detected feature is real (as opposed to pure noise), either based on the measured values or some external information.

When different features are measured with different reliability, subjecting all features to the traditional false discovery rate procedures may yield sub-optimal results. We present two examples here. The first is detecting differentially expressed genes using RNA-seq data. Some genes are measured with low total read counts. For such genes, the measurement reliability, as well as the statistical power of detecting their differential expression is limited. As a result, low p-values cannot be attained when robust testing procedures are used^12^,^13^,^14^. When a false discovery rate procedure is applied to the test results of all genes, the low-read count genes mostly contribute to the null (non-differentially expressed) distribution. Involving both high-read count and low-read count genes in the FDR or lfdr procedure will reduce the significance level of all the genes. Wu *et al.*^15^ recently showed that excluding genes with low read count greatly improves the power in differential expression analysis of RNA-seq data.

The second example is more extreme. In LC/MS metabolomics data, features are detected based on the data point patterns in the three dimensional space of mass-to-charge ratio (m/z), retention time (RT) and signal intensity^16^,^17^. Certain signal to noise ratio (S/N) threshold and peak shape models are applied. The number of features detected relies on the stringency of the peak detection criterion. There is a trade-off between mistaking noise as features versus losing of true features with low intensities^18^. Often lenient thresholds are used in order to capture as many real features as possible, and as a result, a large number of features are detected. Presumably some of them are derived from pure noise. The hope is that such features will be filtered out in the down-stream feature selection process. However, the presence of such false features reduces the significance of all the features in false discovery rate calculation. This is compounded by the fact that we can never know how many false or noise features there are in real datasets.

A simple illustration of this issue is presented in Fig. 1. In this simple simulation, we demonstrate the effect of the existence of pure noise features on the FDR adjustment. We simulated p-values of non-differentially expressed features from the uniform distribution, and the p-values of differentially expressed features from a Beta(1,100) distribution. We applied two widely used FDR approaches – the Benjamini-Hochberg procedure^1^, and the Storey q-value procedure^2^. As shown in Fig. 1a, when no pure noise genes are present, approximately 400 features are claimed significant at the FDR level of 0.2, which is close to the hidden truth (Fig. 1a). However when pure noise features are present, they contribute to the null distribution, i.e. the uniform distribution in this case, and make all features less significant. In this case, less than 100 features can be claimed significant at the FDR level of 0.2 (Fig. 1b).

Although the involvement of pure noise features is an extreme scenario which is only relevant in some metabolomics data, similar effects can be caused by features measured with low reliability, e.g. low read count genes in RNA-seq data, probesets with highly variant probe intensities in microarray data, and proteins with few matched peptides in proteomics data. Such varying reliabilities can impact lfdr estimation as well. We illustrate this point using simulated data with additive noise (Fig. 2). When the noise level of a feature is high, even if it is differentially expressed, its test statistic is likely to fall close to the center of the null distribution (Fig. 2a, red points in the upper region). Thus in traditional lfdr estimation, not only is the feature unable to be detected as differentially expressed, but it also contributes to the null distribution, making the detection of other differentially expressed features more difficult. This is seen in Fig. 2b–e, which are obtained from Efron’s lfdr procedure^5^. Comparing Fig. 2b,d, we see that when all features are considered, the distribution of the truly differentially expressed features overlap substantially with the null distribution. When we only consider the more reliable features (Fig. 2d), the two densities separate better. Correspondingly, if we use all features in lfdr estimation (Fig. 2c), a more stringent threshold is estimated (yellow triangle in Fig. 2c), as compared to only using more reliable features in lfdr estimation (Fig. 2e). Although stratification by reliability score is not actually conducted in data analysis, it achieves a similar effect as using two-dimensional densities derived from both the test statistic and the reliability score to estimate the lfdr, which we advocate in this manuscript. As indicated by Fig. 2a, the purpose is not to capture differentially expressed features measured with high noise, but rather to improve the lfdr estimates of differentially expressed features measured with low noise.

Often the reliability of features can be partially quantified, not necessarily in rigorous statistical terms, but with good heuristic approximation that makes intuitive sense. In this study, we focus on metabolomics data, which has one of the most severe feature reliability issues among all omics data types. Several quantities can be used to indicate how reliable a metabolic feature is. They include the percentage of missing values, the magnitude of the signal, and within-subject variation when technical repeats are available. In this study, we propose a composite reliability index for metabolomics data. Once the reliability is quantified, we devise a new lfdr procedure to incorporate reliability for better lfdr estimation. In simple terms, the method amounts to a soft stratification of features based on their reliability levels. Each feature is compared to the null distribution derived from all the features with similar reliability level to obtain the lfdr values. The null density is computed based on permutation without changing the reliability indices. Our estimation procedure bears some resemblance to the multi-dimensional lfdr by Ploner *et al.*^8^. However it is different in two major aspects. First, the Ploner method is devised to address the well-known issue that features with small standard errors are more likely to be false discoveries. The logarithm of standard error is used in conjunction with t-test statistic^8^. The same issue was also addressed in 1-dimensional lfdr correction by adding a constant to the standard error term to generate a modified t-statistic^19^. In the situations we consider, the feature reliability measures the technical variation alone, which is independent of the test statistic. Thus it can be applied on top of the 1-dimensional modified t-statistic^19^ that already partially addresses the small standard error issue. Secondly, we propose a new robust estimation procedure for the null density. Two-dimensional (2D) density estimation using nonparametric methods requires dense enough data points for reliable estimation. It is not stable at the regions with few observations, which also happen to be critical regions for the lfdr estimation. Given the measure of feature reliability is independent from the test statistic, we propose estimating the 2D density of the null distribution using the product of the two 1D densities of the test statistic and the reliability score. This estimation is more reliable than directly estimating the 2D densities at regions where points are sparse.

## Methods

### The local false discovery rate procedure

Following the consensus of the lfdr literature, we consider the density of the test statistic:


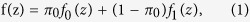


where *f* is the mixture density for the observed statistic 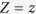, 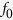 and *f*_1_ are the respective densities of the test statistic of the null (non-differentially expressed) and non-null (differentially expressed) features, and *π*_0_ is the proportion of true null features.

The lfdr is then defined as


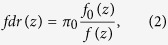


at observed test statistic *Z* = *z* and *z* is a *k*-dimensional statistic.

In this study, the test statistics were obtained from a metabolome-wide association study (MWAS). To identify metabolic features whose expression levels are associated with a certain clinical outcome or risk factor, simultaneous hypothesis testing is carried out. We used linear models with log-intensity of the features as the dependent variable, and the risk factor as the independent variable, adjusting for other confounders, e.g. age, gender, ethnicity and experimental batch effect. The regression is conducted one metabolic feature at a time. After obtaining the t-statistic and the corresponding p-values of all the metabolic features, different FDR and lfdr procedures can be applied to select significant metabolic features.

Here, we compare the 1-dimensional (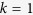; fdr1d) and 2-dimensional (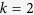; fdr2d) lfdr procedures. Density estimation is done non-parametrically. The fdr1d only uses the t-statistic (*z* = *t*) from the simultaneous hypothesis testing, while fdr2d takes both 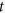 and the reliability index 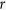 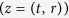, which will be further described in the next sub-section. Estimation of (2) is done via plug-in estimators of *π*_0_, *f*_0_(*z*) and *f*(*z*).

Let the observed statistics be denoted as 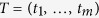 and 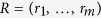, where *m* is the number of metabolic features. The null density *f*_0_(*z*) is estimated using the permutation method. We permute the risk factor (independent variable of the MWAS analysis) to obtain 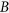 sets of permuted variables. We run the MWAS procedure described earlier using each set of the permuted risk factor as the independent variable. The 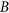 sets of t-statistics 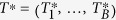 produced from the MWAS form the dataset for non-parametric estimation of 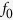. In this study, we used *B =* 10.

For fdr1d, both *f*_0_ and *f* are estimated using kernel density estimation methods, available in the R package KernSmooth^20^,^21^. The bandwidth is selected using existing direct plug-in methodology^20^,^22^. The observed density *f* is estimated using the observed data *T*.

For fdr2d, the null density *f*_0_ is estimated using the permuted dataset, 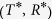, where 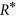 is just *K* replicates of *R*, as the reliability scores do not change in the permutations. We allow the estimation to be done in two ways. The first is directly estimating the 2D density using the kernel smoothing method^20^,^21^. The second is first estimating the 1D densities *f*_0,t_ and *f*_0,r_ separately using the permuted data, and then compute the value for each metabolic feature using the product of the two 1D densities,


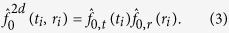


This estimation procedure is only for the estimation of the null density. It is valid because under the null hypothesis of no differential expression, the distribution of test statistic is independent of the measurement reliability. When there is reason to believe the assumption does not hold, we can always fall back to the 2D density estimation. The 2D observed density *f* is estimated using kernel density estimation^20^,^21^, the same method as the first estimation procedure for the null density *f*_0_.

We suggest that π_0_ be estimated from (1), using the estimate obtained from Efron’s 1D lfdr procedure^5^, which is more robust than basing the estimation on a 2D model fitting. In the presence of low-reliability features, the estimate of 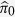 using the 1D approach is an over-estimate of the truth, because low-reliability features only contribute to the null density. Using this over-estimate will result in slightly inflated lfdr estimates, which causes the overall lfdr procedure to be relatively conservative. However this inflation is minor. For example, an increase of 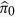 from 0.8 to 0.9 inflates the lfdr estimate by a factor of 1.1, which is well acceptable. With the three estimates 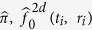 and 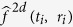, we can plug in these estimators into (2) to get fdr2d.

### The repeat reliability index (RRI) for metabolomics data measured in replicates

Due to the high noise level and low cost of single LC/MS measurements, metabolic intensities are often measured in replicates, i.e. for each feature, there are multiple readings per subject. In each LC/MS profile, a zero value could be observed for a metabolic feature. It can mean the feature is truly absent from the sample, or the measurement is missing due to ion suppression and other mechanisms. Because of the uncertainty, a heuristic approach is commonly taken – if the feature is consistently zero across the replicates, it is considered absent from the sample. If the feature has zero values in some of the replicates, the average of the non-zero measurements are taken as its intensity, which is an implicit missing value imputation process.

In an experiment with *M* replicates, let 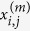 be the log-transformed value of the 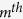 metabolite in the 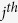 repeat of the 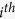 sample. The average measurement of the 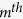 metabolite in the 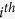 sample is commonly calculated by the mean of non-zero values in the repeats:


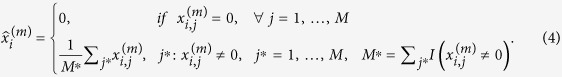


where *I(A)* is the indicator function which takes the value of 1 if *A* is true and 0 otherwise. Accordingly, the reliability index aims at accounting for within-subject variation. It is calculated from those samples where the feature is detected more than once in the repeats:


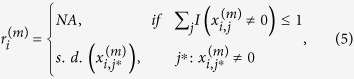



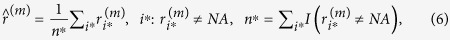


where 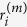 is the standard deviation within each 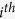 sample and 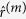 is the reliability index for each metabolite, which is the average of the each non-NA sample standard deviation. By this definition, we have a reliability index that takes smaller values when a feature is more reliably measured. For a feature in a sample, it is possible that the sample has no or only 1 observation out of the 3 replicates. In such cases, the variation is unquantifiable and we assign NA to such features in that sample. Thus, the reliability index of a feature only takes into account standard deviation measures from samples that have more than 1 observed value for the feature.

The smallest possible value of the reliability score is zero, representing the most reliable features. However, there is no upper bound and the top 1% unreliable features (with corresponding reliability scores higher than 99% of the scores of all features) can spread across a wide range. Since some extremely high reliability scores can cause difficulty in two-dimensional density estimation, we compress the reliability scores at the 99^th^ percentile to make the computation more robust. That is, for all reliability indices greater than the 99^th^ percentile, we replace their value with the 99^th^ percentile, which is the reliability index value that is larger than 99% of the other values. The 99^th^ percentile is used as a cutoff to deal with outlying metabolites with extremely high variation in their measurements, without affecting the main cluster of reliability indices and has little impact on the more reliable features.

## Results

### Simulation study

We simulated the log-scale expression data of 8000 metabolites in 100 samples (50 control samples and 50 disease samples) with triplicate measurements. We generated the data using the following procedure:

1. We first generated the true expression levels of 5000 real metabolites using multivariate normal distribution with a variance-covariance structure extracted from a real dataset. The median standard deviation was 1.6. The first quartile of the standard deviation is 1.3, and the third quartile is 1.9 (Interquartile range = 0.6).
2. Each column of the data was repeated three times.
3. Additive noise was added to the data at various levels. The maximum level of noise standard deviation (SD) was specified to be 2.5. For each metabolite, we took a random number from the uniform distribution between zero and the maximum noise SD value. Using this value as the SD, we generated Gaussian white noise and added to the expression levels of the metabolite.
4. A pre-specified number of metabolites were randomly selected to be differentially expressed. A pre-specified signal level was added to the disease samples for these metabolites.
5. The expression levels of another 3000 pure noise metabolites were generated from the normal distribution with a standard deviation that equals the maximum noise SD as in step (3). This pure noise matrix was combined with matrix of the 5000 real metabolites.
6. The number of zero measurements in each row was drawn from an exponential distribution with a rate parameter of 1/30. Once the number of zeroes was determined for a metabolite, the zero entries were randomly selected in the row, and the corresponding positions in the matrix was assigned a value of zero.

We ran our simulations using 5 different true signal strengths – 0.75, 1, 1.5, 2, 2.5 and 4 different levels of differentially expressed metabolites – 100, 200, 300, 500. For each simulation setting, i.e. combination of true signal strength and the number of true differentially expressed metabolites, we simulated the data 10 times. Once the data was generated, we analyzed it by first generating the t-statistic of every feature, and then estimating lfdr using five different procedures: the fdr2d procedure, the fdr2d with independence assumption, the 2D lfdr procedure using standard error by Ploner *et al.*^8^, the fdr1d procedure, and the locfdr package^5^. At the lfdr threshold of 0.2, each method identified a number of metabolites as differentially expressed. This list was compared with the list of truly differentially expressed metabolites. The methods were compared based on the True Positive Rate (TPR): the percentage of truly differentially expressed metabolites called significant, and the False Discovery Rate (FDR): the percentage of metabolites called significant that were in fact not differentially expressed.

Figure 3 shows the simulation results. The permutation-based fdr1d method generated very similar results as locfdr. Thus we only show the locfdr results in Fig. 3. Each subplot represents a scenario of number of differentially expressed features. When the number of differentially expressed features is low (upper-left panel), at lower signal levels, all the three 2D density-based methods yielded FDR slightly over 0.2 (red, green, and blue dashed curves). Given the overall low count of true differentially expressed metabolites, and the low count of metabolites called significant, a slight bias towards sensitivity is tolerable. In other situations, the FDR levels are mostly correctly controlled by all the methods. The fdr2d based on independence assumption (red curves) showed highest power (TPR) while controlling FDR at similar levels with the other methods. Its performance is followed by the fdr2d using 2D density (green curves). The 2D method using standard error (Ploner’s method) trailed the two methods except when the signal strength is very low (blue curves). At the same time, all three 2D methods generally achieved better statistical power than the 1D method (grey curves). Although the 1D method achieved the lowest FDR levels, it is clear that the control is overly conservative, causing unnecessary loss in the discovery of truly differentially expressed metabolites. With the increase of the number of truly differentially expressed metabolites and/or signal strength, the difference between the methods became smaller. Still the same trend persisted.

As the fdr2d based on independence assumption (red curves) and the fdr2d using two-dimensional density estimation for the null (green curves) only differ by their estimation procedure of the null density, the performance difference is entirely due to the estimation procedure. In our simulation settings, the reliability score is truly independent from the test statistics. As the two-dimensional density estimation is less robust in regions with few data points, using the product of two one-dimensional densities generated more reliable results.

We conducted another set of simulations in which no metabolite is differentially expressed (null experiment). Out of 5000 simulated metabolites, the permutation-based fdr2d procedure generated an average of 8.5 false positives, the fdr2d method with independence assumption generated an average of 4.0 false positives, the fdr1d method and the locfdr package both generated an average of 0.5 false positives. Although the 2D methods generated a few more false positives under the null situation, the false positive rate is still well within control. The maximum false positive rate is 1.7 × 10^−3^. Thus the new methods are not achieving higher statistical power at the expense of excessive false positives.

### Real data analysis

In this study, we used the metabolomics data generated from 494 subjects from the Emory Cardiovascular Biobank, which consists of patients who have undergone coronary angiography to document the presence/absence of coronary artery disease (CAD). Demographic characteristics, medical histories, behavioral factors and fasting blood samples have been documented and details about risk factor definitions and coronary angiographic phenotyping have been described previously^23^,^24^. Each sample was analyzed in triplicate with high-resolution liquid chromatography – mass spectrometry (LC-MS), using anion exchange and C18 chromatography combined with the Thermo Orbitrap-Velos (Thermo Fisher, San Diego, CA) mass spectrometer using an m/z range of 85 to 850. The data was pre-processed using xMSAnalyzer^25^ in combination with apLCMS^17^,^26^. For each feature, there were three readings per subject. An average metabolite intensity value was calculated from the non-zero readings for each individual. That is, an average reading of 0 was obtained only if all 3 readings for the individual were 0. This is the combined metabolite data we used for subsequent analysis. In our analysis, batch effect was accounted for linearly in the MWAS regression analysis as a confounder. There were 18,325 metabolic features detected.

In this proof-of-concept study, the risk factor of interest was High-Density Lipoprotein (HDL), the levels of which is known to be inversely associated with the risk of cardiovascular disease. The HDL levels ranged from 5–95 mg/dL, with mean 42.3 mg/dL and standard deviation 12.8 mg/dL. The reliability indices of each feature of this dataset of 18,325 features were calculated as described in the methods section. They ranged from 0.0417–1.044, with smaller values indicating more reliable measurements. The 10% most reliable features (*n* = 1837) had reliability indices of 0.0417–0.185.

Using our proposed fdr2d method, we found 384 significant features at the lfdr cutoff of 0.2, while fdr1d found 139 significant features. Between the two lists, 108 of the significant features overlap, which means most of the features found by fdr1d were also found by fdr2d, and fdr2d found an extra ~240 features. We used pathway analysis to determine whether the selected features were biologically meaningful. We used *mummichog* Version 0.10.3^27^ to conduct pathway analysis and possible metabolite identification.

Figure 4(a,b) show the significant pathways indicated by *mummichog* for the fdr2d and fdr1d methods respectively. The significant pathways common to both the fdr1d and fdr2d methods include urea cycle/amino group metabolism, purine metabolism, and drug metabolism – cytochrome P450. These pathways will be examined briefly next.

The urea cycle takes place mainly in the liver for mammals and since HDL is synthesized in the liver, it is not surprising that urea cycle/amino group metabolism pathway is found significant. The cholesterol delivered to the liver is secreted into the bile after conversion to bile acids, and it is interesting that the fdr2d method has also detected this pathway (bile acid biosynthesis). Hepatocyte nuclear factor-1alpha (TCF1) regulates both bile acid and HDL metabolism^28^.

Another common pathway in both analyses is purine metabolism. Studies have shown that increased levels of uric acid, the end-product of purine metabolism, are associated with decreased HDL cholesterol, and increased risk of cardiovascular events^29^,^30^,^31^.

Cytochromes P450 (CYPs) are major enzymes involved in drug metabolism. Expression of CYP has been linked with increased levels of HDL cholesterol^32^. In addition, the class B type I scavenger receptor, SR-BI, is an HDL receptor that provides substrate cholesterol for steroid hormone synthesis^33^ and SR-B1transgenic mice have shown decreased levels in some CYP enzymes^34^.

Overall, more pathways were identified under the fdr2d method. Most of the additional pathways were associated with lipid metabolism, such as glycosphingolipid metabolism, glycosphingolipid biosynthesis, Omega-6 and Omega-3 fatty acid metabolism. This makes biological sense since the risk factor in this study is the level of High-Density Lipoprotein (HDL) and HDL particles are responsible for transferring fats away from cells, artery walls and tissues body-wide, ultimately to the liver for other disposal. It has been hypothesized that HDL is a mediator of glycosphingolipid transport and synthesis^35^. A meta-analysis of 60 studies showed that the substitution of mainly omega-6 fatty acid for carbohydrates had more favorable effects on the ratio of total to HDL cholesterol^35^,^36^. In addition, a systematic review indicated significant positive association between consumption of fish oil and alpha-linoleic acid (omega-3 fatty acid) and HDL cholesterol^37^, while another study showed that low intakes of omega-3 fatty acid supplements in bovine milk increase HDL concentrations in healthy subjects^38^.

The lysine metabolism pathway was the most significant in the fdr2d analysis and it has been shown that lysine residues play an important role in HDL metabolism^39^. Other pathways included carbohydrate metabolism and the carnitine shuttle, which is responsible for transferring long-chain fatty acids across the inner mitochondrial membrane and a variant of one of the enzymes involved in the process has been positively associated with HDL cholesterol^40^.

It appears that the pathways indicated by the fdr2d method show the complexity and interconnectedness of HDL and its effect in the human body, which may not be indicated by the fdr1d method, which shows some pathways involved in metabolism of compounds found in HDL. More detailed biological interpretation will be conducted in a separate manuscript that focuses on the biomedical aspects of the Biobank metabolomics data.

We applied Efron’s locfdr function to the real dataset to compare with our lfdr results. There were 161 significant features (lfdr values < 0.2). These features were mostly overlapped with the 139 features significant under the fdr1d method (112 overlap). Pathway analysis revealed similar results to the fdr1d analysis, with the same common pathways as the fdr2d analysis. Additional pathways were also related to compounds of HDL, similar to the fdr1d results. The full list of significant pathways for the locfdr analysis is included in the Supplementary Figure S1. We also looked at the top 10% most reliable features (features with reliability indices less than 0.185, *n* = 1837). Of this subset of features, 179 had raw p-values less than 0.01. Applying Benjamini-Hochberg’s FDR correction^1^ yielded 282 significant features. Pathway analysis for the 179 significant features (full list in Supplementary Figure S2) revealed a mixture of pathways from the fdr1d and fdr2d methods, as well as the butanoate metabolism pathway, which is a fatty acid. On the other hand, pathway analysis for the 282 significant features (full list in Supplementary Figure S3) contained the same common pathways with fdr1d and fdr2d analysis – cytochrome P450 metabolism and purine metabolism. It also had pathways common with fdr2d analysis (bile acid biosynthesis, carnitine shuttle, drug metabolism – other enzymes, lysine metabolism, glycosphingolipid metabolism), as well as other fatty acid biosynthesis and metabolism pathways.

Often jointly studied with HDL is the low-density lipoprotein (LDL). LDL itself is not measured by the LC/MS data, because LC/MS metabolomics measures small molecules. Nonetheless, because LDL was measured by a traditional method in this study, we tried to add it to the metabolite table and conduct the analysis. In our analysis, we treated LDL as a pseudo-metabolite and assigned a reliability score that equaled the lowest observed reliability score of 0.0417 in our data. After obtaining its test statistic from the linear model and adjusting it together with all other metabolites, we found that LDL is significant by the fdr2d method, with an lfdr value of 0.124. On the other hand, the fdr1d method assigned it an lfdr value of 0.603. Because HDL and LDL are known to be associated with heart disease risk in a reverse manner in the population under study, this serves as a positive control and validates our new method.

## Discussion

In the fdr2d approach, the permutation procedure ensures that the reliability index values of the metabolic features, (*r*_1_, … ,*r*_m_) do not change. Thus the marginal density of the data points on the reliability index axis does not change in the permutations. Only the marginal density of the t-statistic, and conditional densities of t-statistic given the reliability index change. As a result, the t-statistic of each feature is effectively compared to the distribution of t-statistics of all features with similar reliability values. Thus in a sense, the fdr2d approach can be approximated by a 1-dimension FDR estimation in which the features are stratified based on their reliability indices. The real data analysis indicated a similar finding, where the pathways yielded by the 282 significant features (FDR-adjusted from the top 10% reliable features) were very similar to the pathways under the fdr2d method. At the same time, the fdr2d method avoids picking a hard threshold, and may retain some features with moderate to low reliability if they are indeed highly associated with the clinical outcome. Thus, the fdr2d method can be preferable to the soft stratification based on reliability.

In broad terms, variation in the measurements of a metabolite can be dissected into biological variation (diet, diurnal variation, etc.) and technical measurement noise. Metabolites with high biological variance should not be confused with those with high measurement noise. In this manuscript, by reliability we mean technical reliability, i.e. a quantity that reflects technical measurement noise. This is straight-forward to estimate in metabolomics data measured with replicates. When replicates are not available, it is possible to derive a reliability score based on other criteria. For example, the goodness-of-fit of peak shape models to the feature, or the percentage of missing intensities in the peak could contain information about how reliable the feature is. We plan to further explore other reliability measures in future studies.

**Figure 2:**
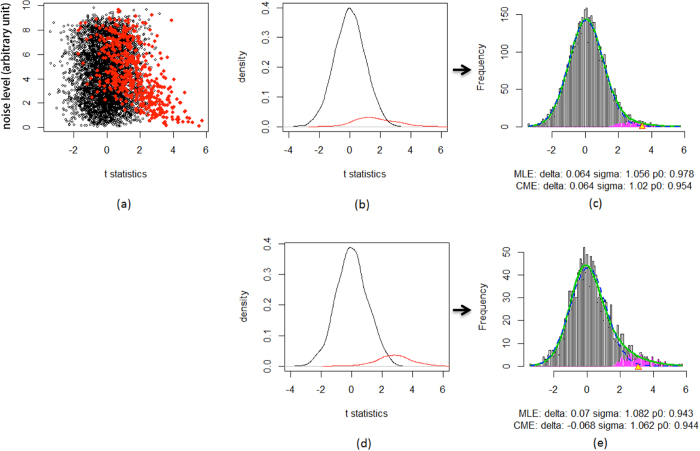
The impact of additive noise on local false discovery rate estimation. The plots are based on simulated data. For simplicity, all the differentially expressed features were over-expressed. (**a**) A two-dimensional (2D) plot of noise level against the t-statistics. Each point represents a feature. Red points: true differentially expressed features. (**b**) One dimensional (1D) density plot of the test statistics of the null features (black) and differentially expressed features (red curve). (**c**) Estimating local false discovery rate from all features using the locfdr package in R^5^. (**d**) 1D plot of the test statistics of the null features (black) and differentially expressed features (red curve), limiting to more reliable features (noise level < 3). This is closely related to the 2D density using both test statistics and reliability scores. (**e**) Estimating lfdr from the more reliable features (noise level < 3). Notice the procedure in (**e**) is not carried out explicitly in data analysis, but it is closely related to estimating lfdr in two dimensions, where each feature is compared to other features of similar reliability level.

**Figure 3:**
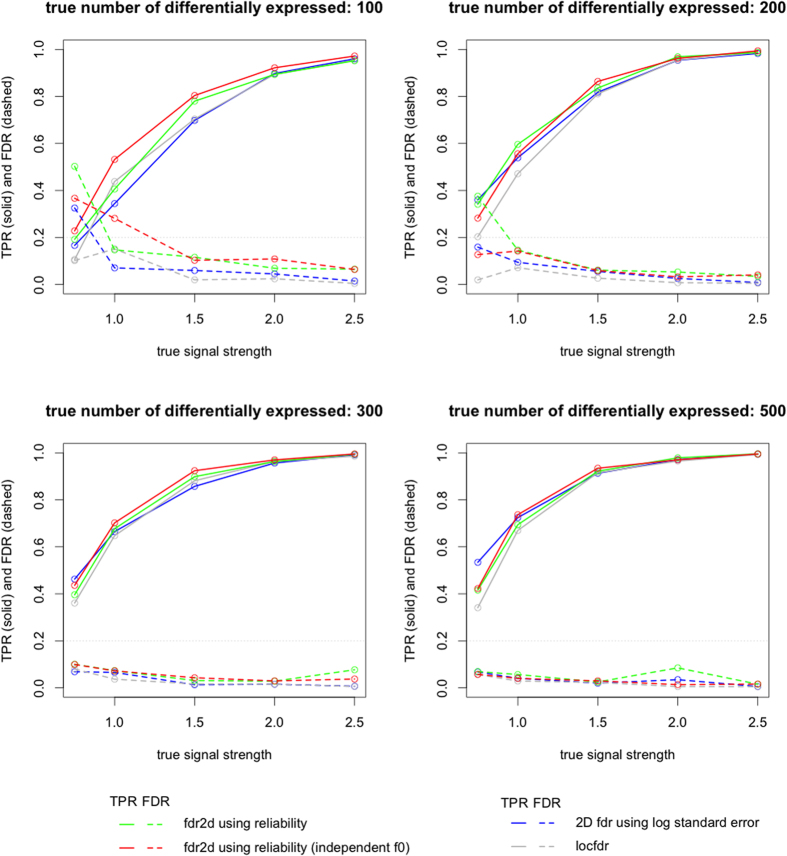
Simulation results. Each sub-plot represents a scenario of number of differentially expressed features.

**Figure 4:**
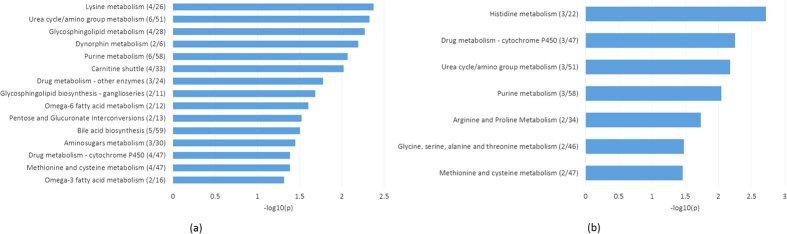
Pathway analysis results using *mummichog*^27^. Important pathways from the significant features identified by the: (**a**) fdr2d method and (**b**) fdr1d method. Numbers in parentheses indicate overlap size/pathway size. Pathway size refers to the number of metabolites out of 18325 were in each pathway. Overlap size refers to the number of significant metabolites that were in each pathway.

The fdr2d approach incorporates the reliability score as a second dimension in accounting for false discoveries. This is in contrast to the fdr1d or other lfdr approaches that only use the t-statistics from the MWAS analysis. As mentioned in the real data analysis in the Results section, the MWAS analysis uses combined replicates, such that there is only 1 intensity value for each subject. The standard deviation involved in the t-statistic calculation accounts for the variation across all subjects (between-subject variation). The fdr2d approach attempts to account for within-subject technical variation by finding the average subject standard deviation for each set of technical replicates. We can then adjust for false discoveries more accurately by placing more emphasis on features with high reliability, as they have more consistent measurement. This is based on the assumption that false features are likely to have inconsistent measurement across technical replicates and hence higher within-subject variation. This was shown in the simulations, when the fdr2d methods were able to detect more truly differentially expressed features than the fdr1d and locfdr methods (Fig. 3). Figure 2a also shows that features that are more unreliable (red points at the top of the figure) tend to cluster around the null and result in less distinction between the distributions of differentially and non-differentially expressed features.

In non-targeted metabolomics, there is a risk of generating a large number of fake metabolites if peak detection is carried out in an overly lenient manner. This is often done when the interest is to detect environmental impacts, because all environmental chemicals exist in the human blood in very low concentrations. When a large number of fake metabolites are present, they certainly contain no biological signal at all. At the same time, their technical variation is very large because they are just noise in LC/MS profiles, hence they have low reliability under our fdr2d method. While traditional methods allow them to contaminate the null distribution, our method suppresses their impact in the statistical inference to obtain more accurate lfdr estimates.

In conclusion, we have presented a method for the computation of lfdr that incorporates reliability index. In situations where substantial noise features are present, the method improves the statistical power of detecting differentially expressed features by minimizing the influence of noise features, because such features tend to have worse reliability values. One major aspect of this procedure is to quantify the reliability using a single variable. As we have shown, a reliability index using average standard deviation among replicates for metabolic features worked well. Similar measures can be derived for other data types.

## Additional Information

**How to cite this article**: Chong, E.Y. *et al.* Local false discovery rate estimation using feature reliability in LC/MS metabolomics data. *Sci. Rep.* **5**, 17221; doi: 10.1038/srep17221 (2015).

## Supplementary Material

Supplementary Information

## Figures and Tables

**Figure 1:**
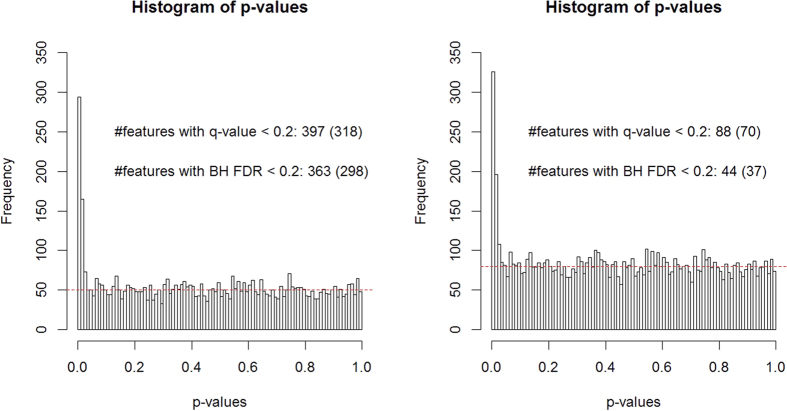
Illustration of the impact of noise features on the calculation of false discovery rate using simulated p-values. p-values of 5000 non-differentially expressed features and 400 differentially expressed features were generated from the uniform distribution and exponential distribution respectively. The histograms of p-values are shown. Red dashed line: the part of the histogram corresponding to the non-differentially expressed genes. Number of differentially expressed features detected in each case are indicated in the parentheses. (**a**) Without noise features. (**b**) With an additional 3000 noise features, whose p-values follow the uniform distribution.
