# Supplementary material for: Local false discovery rate estimation using feature reliability in LC/MS metabolomics data
Source: Sci Rep. 2015 Nov 24;5:17221. doi: 10.1038/srep17221 (PMC4657040; doi:10.1038/srep17221)

Local false discovery rate estimation using feature reliability in LC/MS metabolomics data

Elizabeth Chong [1], Yijian Huang [1], Hao Wu [1], Nima Ghasemzadeh [2], Karan Uppal [2], Arshed A. Quyyumi [2], Dean P. Jones [2], Tianwei Yu[1]*

[1] Department of Biostatistics and Bioinformatics, Rollins School of Public Health, Emory University, Atlanta, GA, USA, 30322

[2] Department of Medicine, School of Medicine, Emory University, Atlanta, GA, USA, 30322

SUPPLEMENTARY MATERIAL

Supplementary Figure S1

S1. Pathway analysis for 161 significant features under Efron's locfdr package. Numbers in parentheses indicate overlap size/pathway size. Pathway size refers to the number of metabolites out of 18325 were in each pathway. Overlap size refers to the number of significant metabolites that were in each pathway.

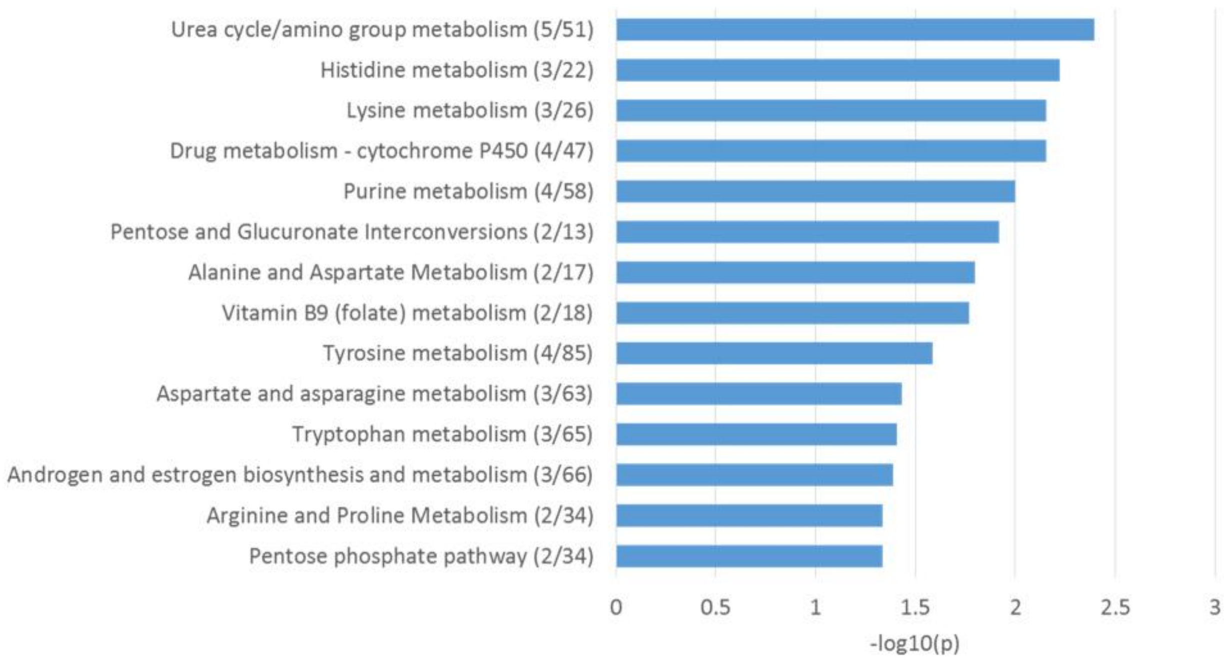

Supplementary Figure S2

S2. Pathway analysis for 179 significant features ($p<0.01$) in the subset of top 10% reliable features (n = 1837). Numbers in parentheses indicate overlap size/pathway size.

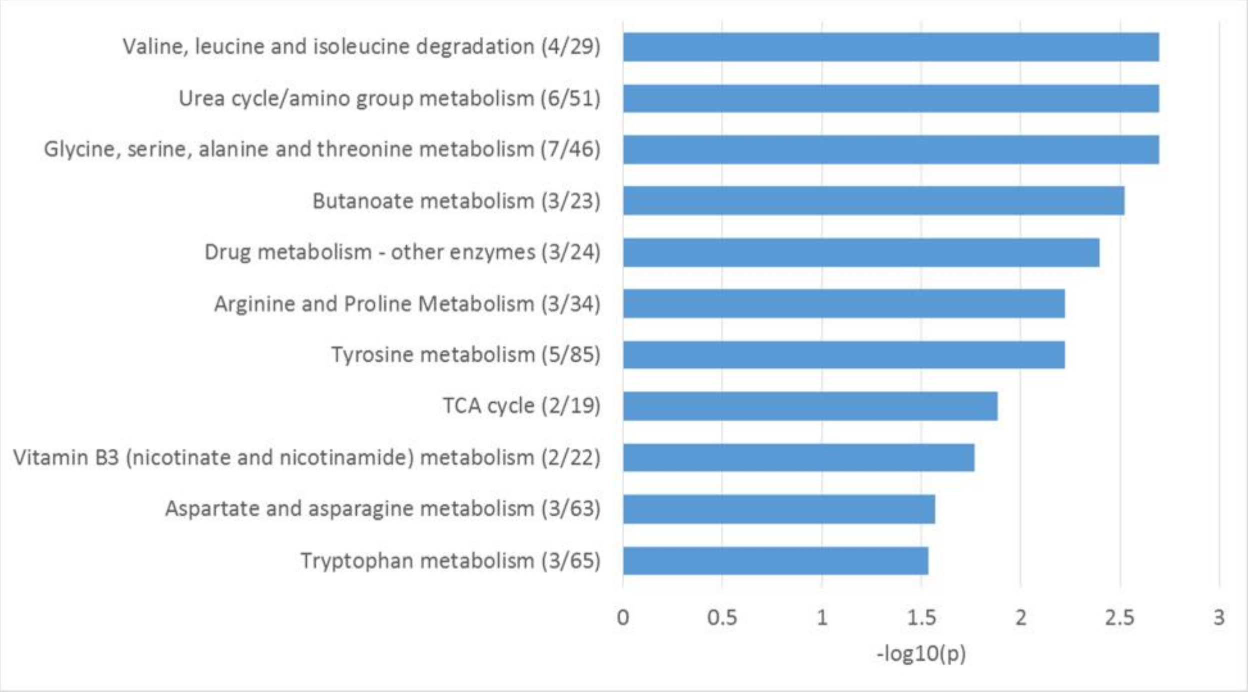

Supplementary Figure S3

S3. Pathway analysis for 282 significant features after Benjamini-Hochberg FDR correction in the subset of top 10% reliable features (n = 1837). Numbers in parentheses indicate overlap size/pathway size.

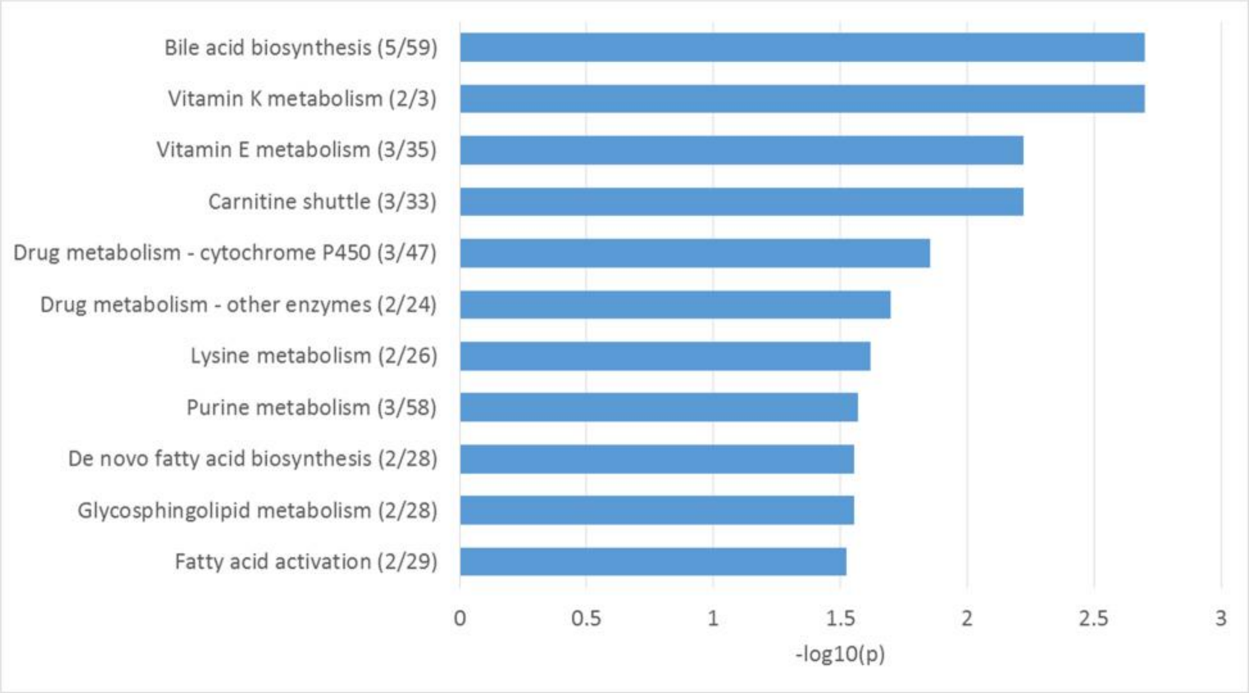

Supplement: Supplementary Information [file srep17221-s1.pdf]
